# Supplementary material for: ESQmodel: biologically informed evaluation of 2-D cell segmentation quality in multiplexed tissue images
Source: Bioinformatics. 2023 Dec 28;40(1):btad783. doi: 10.1093/bioinformatics/btad783 (PMC10783950; doi:10.1093/bioinformatics/btad783)
Supplement: btad783_Supplementary_Data [file btad783_supplementary_data.pdf]

Supplementary Material for Lee et al., ESQmodel: biologically informed evaluation of 2-D cell segmentation quality in multiplexed tissue images

1 Supplementary Note

1.1 Datasets

1.1.1 Simulated datasets

Simulated datasets were generated by forward simulation to assess model performance against three different types of segmentation errors. First, we sampled from our described model. The expression and prior matrix was created by referencing to a labeled human bone marrow mass cytometry dataset [1]. The simulated expression were sampled from Gaussian distributions with their means set to the mean expression profiles of different cell types in the reference dataset. An arcsinh transformation was applied as normalization. Next, we introduced the errors to create each dataset: (1) we built partial segmentation datasets by sampling noise percentage variables to introduce random loss of cell area which have an uneven distribution of marker intensity. As the intensities is the average across all pixels within the cell boundary, this can introduce a different average intensity value depending on the region of the cell area being sampled. We also introduce a minor noise variable to simulate background. The noise percentage is applied to 25% and 50% of cells respectively to create two separate datasets; (2) we built split segmentation datasets by sampling split percentage variables to divide cells in half. This works similarly to (1) however, we split single cells to pairs of cells each with a portion of the original total expression. The resulting intensity is the average of the pixel intensities in the resulting area; (3) we built merge segmentation datasets by randomly merging cells. This is the average of the combined expression between cells with a reduced total cell count. Using this method, we simulated 90 13-marker expression matrices of 2000 cells with a cell type count enumerated from 2 to 10 each with 10 replicates. For each expression matrix, we introduced the three segmentation errors which makes a total of 270 datasets.

1.1.2 METABRIC IMC dataset

36 IMC images from a published breast cancer imaging mass cytometry dataset of the METABRIC cohort [2] were analyzed to evaluate the performance on real data. Raw IMC images and the expression matrix of segmented hyper-stacked images were used.

In-house segmentation was performed by using CellProfiler and Ilastik under a modified procedure of the ImcSegmentationPipeline [3]. Four datasets featuring the well segmented and the three types of segmentation errors—partial, split, and merge—were made through manual labeling using Ilastik. We subsetted the dataset by retaining only selected well-stained immune, stromal, and epithelial markers including CD45, SMA, CK5, and CK7. The original labeled cell types were also reduced to one of the seven labeled cell types: stromal, immune, CK5+, CK7+, SMA+, epithelial, and CK5+ SMA+. A prior expression matrix was generated from the original expression matrix for the seven merged cell types. All generated expression matrices were normalized by an arcsinh transformation with a co-factor of 0.8 and clipped at the 99.5 percentile per marker prior to performing inference.

1.1.3 CHL, RLN, and Tonsil IMC datasets

3 IMC images each of CHL, RLN, and tonsil were generated in-house to compare segmentation accuracy on different tissue types. Procedures for IMC including antibody titration, staining, and acquisition are as previously described [2]. Five representative segmentation methods were performed on the images, including the modified procedure of the ImcSegmentationPipeline, the watershed algorithm, StarDist, Cellpose and DeepCell. Subsetting of the different datasets were done by retaining only selected lineage protein markers for representative cell types. Prior expression matrices and normalization was performed as described in the METABRIC IMC dataset section. An additionally layer of scaling was done on these datasets to assist in performance.

1.1.4 Tonsil MIBI, CODEX, and CycIF datasets

3 datasets were used to address the broad applicability of ESQmodel across different spatial imaging platforms. A tonsil MIBI dataset was downloaded from Ionpath Public MIBItracker (https://mibi-share.ionpath.com/tracker/overlay/sets/97/555). A tonsil CODEX dataset was imaged in-house using the standard experimental approach from the manufacturer. A tonsil CycIF dataset was from Rashid et al. (2019) [4]. Four segmentation methods were performed on the images: the watershed algorithm, StarDist, Cellpose and DeepCell. The modified ImcSegmentationPipeline was not used here as it was only applicable to IMC datasets. Similar to the above datasets, subsetting of the different datasets were done by retaining only selected lineage protein markers for representative cell types. Prior expression matrices, normalization, and scaling was performed as described previously.

1.2 Segmentation Settings

1.2.1 Ilastik Segmentation Settings

We performed segmentation using Ilastik [5] as part of the modified ImcSegmentationPipeline [3]. This modified version [6] of the pipeline allows for inputs of OME-TIFFs directly on top of MCD or TXT files which are not available for some public datasets, such as the METABRIC IMC dataset [2].

The pipeline utilizes a combination of CellProfiler [7] image processing scripts and a user created Ilastik image segmentation project. The CellProfiler scripts can be run as given with small modifications to the parameters, such as the number of markers. The procedure to setting the parameters are detailed in the README file of the repository of the modified pipeline. The Ilastik segmentation project needs to be created by the user. The procedure to segment the cells depend on the markers available in the panel. We detail the markers used for each dataset below.

- METABRIC breast cancer dataset (BC): We used Ir191 and Ir193 DNA intercalators to identify the nucleus. Cytokeratins (CK5, CK7 and panCK) were used to identify the cytoplasmic regions.
- Classical Hodgkin's lymphoma dataset (CHL): We used Ir191 and Ir193 DNA intercalators to identify the nucleus. For membrane segmentation, we used the membrane markers from the IMC cell segmentation kit (TIS-00001) from Fluidigm. CD30 was used to identify Hodgkin and Reed-Sternberg cells.
- Reactive lymph node dataset (RLN) and human tonsil dataset: We used Ir191 and Ir193 DNA intercalators to identify the nucleus. For membrane segmentation, we used the membrane markers from the IMC cell segmentation kit (TIS-00001) from Fluidigm.

To introduce the different segmentation errors in Ilastik, we used different strategies to train Ilastik to perform erroneous segmentation. For merge segmentation, we merged two nuclei together and filled in the cytoplasm that surrounds the two nucleus. For partial segmentation, we filled in only parts of the nucleus and drew the cytoplasm as a thin layer outside of the nucleus. For split segmentation, we split the nucleus into half.

1.2.2 Watershed Segmentation Settings

We ran the watershed algorithm for cell detection in QuPath [8]. As a pre-processing step, the IMC converter [9] was used to convert MCD files to OME-TIFFs. Cell detection relies on a nuclear stain for each dataset. For all datasets, we used the Ir193 DNA intercalator. The parameters set are the following: we set the minimum area to 5 μm^2 and cell expansion was set to 2μm, intensity threshold was set to 5. The parameters were consistent across all images.

1.2.3 StarDist Segmentation Settings

We also employed StarDist [10] for cell detection in QuPath. As a pre-processing step, the IMC converter was used to convert MCD files to OME-TIFFs. StarDist is similar to watershed as it only depends on a nuclear stain and we also used the Ir193 DNA intercalator for each dataset. For our experiments, we used single channel pre-trained models for StarDist that were developed by the StarDist developers: dsb2018_heavy_augment.pb and dsb2018_paper.pb [11]. The model that performed relatively better on our datasets was termed as the regular StarDist while we termed StartDist-Poor as StarDist used with the alternative model that had poorer performance.

1.2.4 Cellpose Segmentation Settings

We also introduced Cellpose [12] for cell detection in QuPath. OME-TIFFs were directly used in QuPath where the procedure is similar to StarDist but requires a QuPath Cellpose/Omnipose extension (https://github.com/BIOP/qupath-extension-cellpose). For our experiments, we used the default settings in the detection script.

1.2.5 DeepCell Segmentation Settings

We also used DeepCell Mesmer [13] for cell detection in QuPath. OME-TIFFs were directly used in QuPath where the procedure is similar to StarDist but requires an ImageJ Plugin for interacting with the DeepCell Kiosk (https://github.com/vanvalenlab/kiosk-imageJ-plugin). For our experiments, we pre-processed all tiffs to generate new tiffs with only the nuclear stain and a cytoplasmic marker to satisfy the requirements of Mesmer. The tiffs were used for cell boundary detection in ImageJ where the overlay was transferred back to the original tiffs.

1.3 METABRIC IMC Subset

To demonstrate performance of ESQmodel, we used a random subset of the METABRIC IMC dataset. We list the 36 patient image codes: MB0005_1_211, MB0064_1_152, MB0109_1_487, MB0120_1_462, MB0135_1_320, MB0150_1_155, MB0199_1_157, MB0221_1_105, MB0237_1_247, MB0254_1_257, MB0272_1_285, MB0301_1_287, MB0307_1_106, MB0309_1_551, MB0312_1_178, MB0321_2_412, MB0324_1_455, MB0344_1_363, MB0354_1_36, MB0370_1_275, MB0390_1_243, MB0392_1_280, MB0394_2_417, MB0416_1_224, MB0470_1_94, MB0530_1_348, MB0549_2_547, MB0568_1_204, MB0573_1_352, MB0583_1_133, MB0594_1_498, MB0601_1_271, MB0622_1_283, MB0623_1_4, MB0646_1_506, MB0904_1_410.

For the breast cancer datasets used for comparative entropy analysis across segmentation methods, we used the following 10 images in order: MB0321_2_412, MB0005_1_211, MB0646_1_506, MB0254_1_257, MB0312_1_178, MB0120_1_462, MB0324_1_455, MB0601_1_271, MB0594_1_498, MB0392_1_280.

2 Supplementary Figures

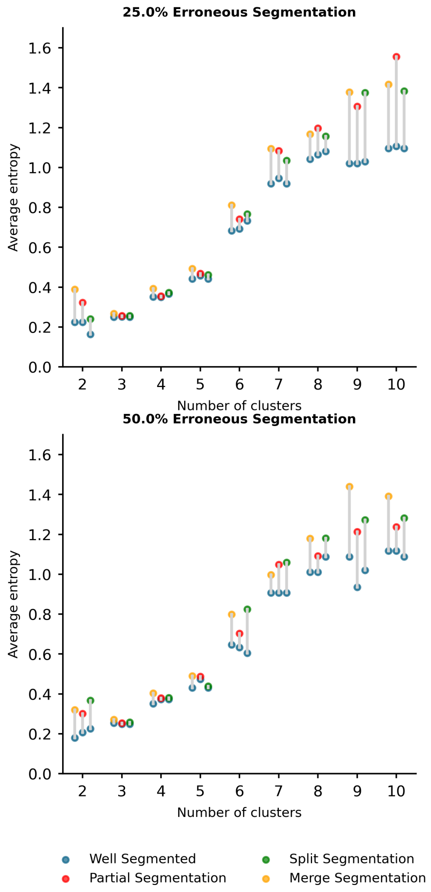

Fig. 1 Performance on simulated data. Dumbbell plots of change in average entropy between well segmented and three cases of erroneously segmented expression data are shown. Two sets of data were created with (**a**) 25% and (**b**) 50% of total amount of cells being erroneously segmented. Colors represent different segmentation datasets.

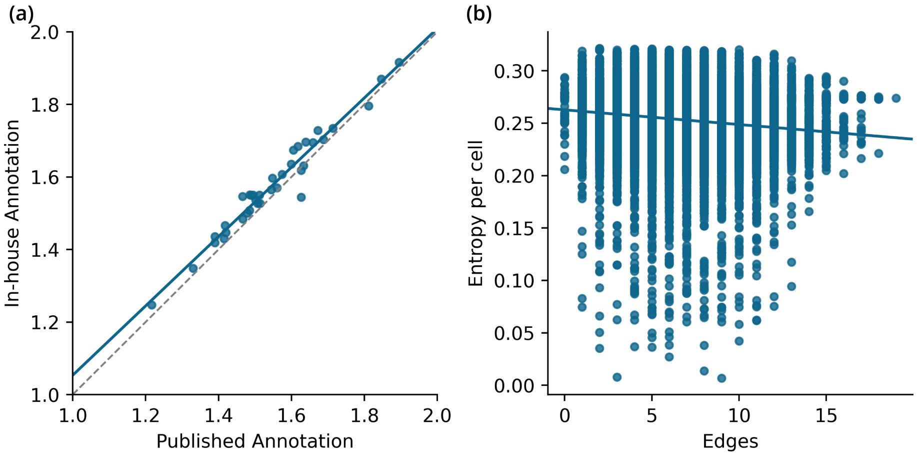

Fig. 2 (**a**) Correlation plot between the published annotation and in-house annotation of the METABRIC IMC dataset. (**b**) Correlation plot between the number of edges per cell and cellular entropy.

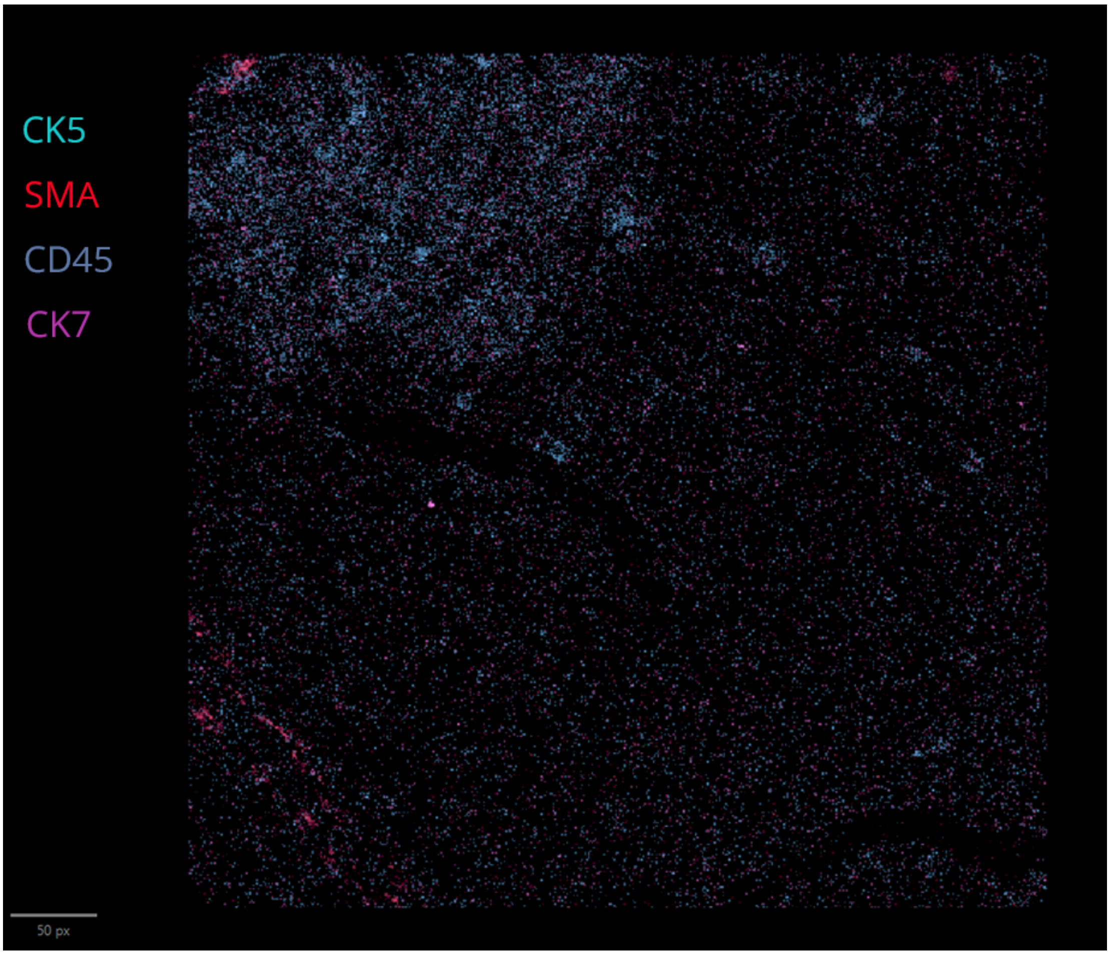

Fig. 3 The IMC multicolored image of the exemplar sample in Figure 3. CK5, SMA, CD45, and CK7 are colored in cyan, red, blue, and purple respectively.

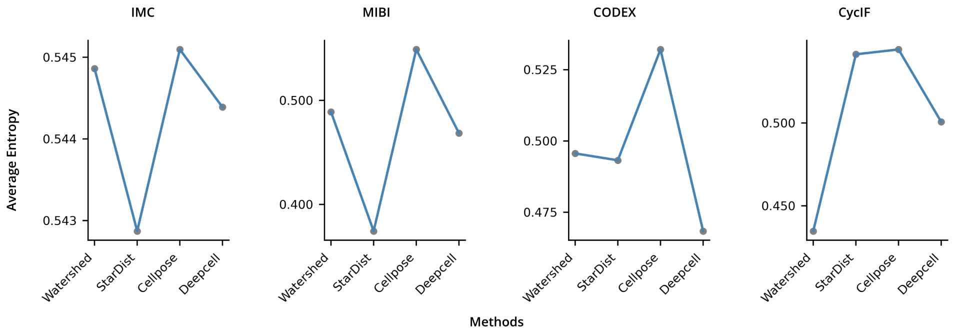

Fig. 4 Comparison of different segmentation methods on tonsil imaged across different spatial imaging platforms. Line plots of the average entropy for each ROI under four different segmentation conditions.

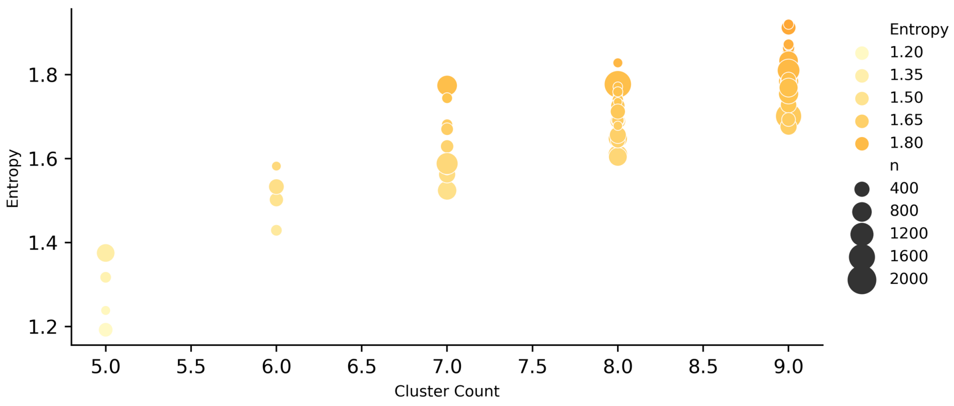

Fig. 5 Distribution of average entropy per cluster count of the METABRIC IMC dataset. Each dot represents a segmented and processed IMC image. The cluster counts are assigned to each image as annotated in the original publication data. An increase in color gradient indicates greater average entropy. An increase in the size of dots indicates greater cell count in the images.

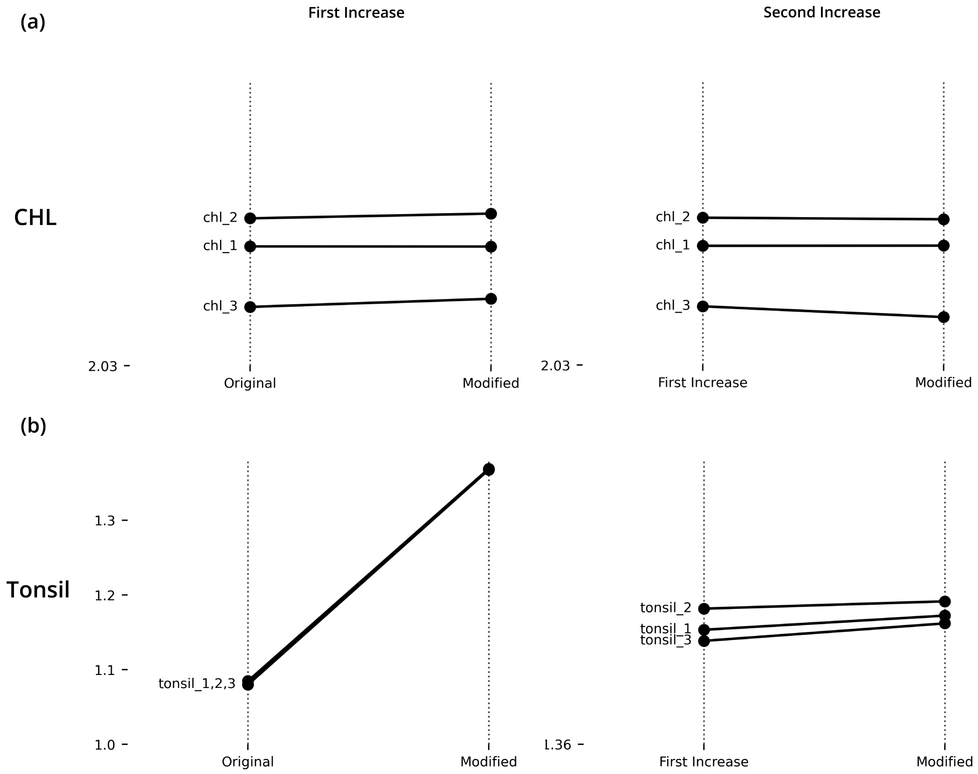

Fig. 6 Comparison of entropy with more markers being introduced. The first increase of CHL included two extra markers: CXCL12 and CXCR3 where tonsil included one extra marker: CD68. The second increase was including four extra markers: Fibronectin, Granzyme B, Vimentin, Galectin9, and dropping CXCR3 where tonsil included an extra BCL2. Original indicated the original markers where CHL had "CD4", "CD30", "CD20", "CD8a", "CD11c", "CD45RO" and tonsil had "CD20", "CD3".

3 Supplementary Tables

3.1 Supplementary Table 1. Scores of entropy from ESQmodel and various existing segmentation metrics to complement segmentation quality assessment for Figure 4.

Batch	Entropy	Cell Count	Average Cell Size	Cell Coverage by Hundred Pixels	Cell Foreground Coverage	Cell Background Coverage	Cell Mask Foreground Coverage	Cell-Nuclei Match
breast_1+Ilastik	0.387472116	1429	128.78	0.60	0.99	0.00	0.99	0.93
breast_1+Ilastik_bad	0.392234185	1536	63.64	0.64	0.78	0.22	0.78	0.99
breast_1+StarDist	0.446158739	1006	169.24	0.42	0.99	0.00	0.99	0.65
breast_1+StarDist_bad	0.446485146	761	189.44	0.32	0.99	0.00	0.99	0.50
breast_1+Cellpose	0.445230297	1020	107.00	0.43	0.87	0.13	0.87	0.66
breast_1+Deepcell	0.445631597	914	106.59	0.38	0.78	0.22	0.78	0.60
breast_1+Watershed	0.446094684	840	114.60	0.35	0.77	0.23	0.77	0.48
breast_2+Ilastik	0.448943576	1377	130.49	0.63	0.99	0.00	0.99	0.79
breast_2+Ilastik_bad	0.472961505	1737	38.23	0.80	0.59	0.41	0.59	0.99
breast_2+StarDist	0.443853662	1199	122.73	0.55	0.99	0.00	0.99	0.69
breast_2+StarDist_bad	0.443581497	1031	130.93	0.47	0.99	0.00	0.99	0.59
breast_2+Cellpose	0.445360223	1067	86.95	0.49	0.82	0.18	0.82	0.61
breast_2+Deepcell	0.445315418	776	79.51	0.36	0.55	0.45	0.55	0.45
breast_2+Watershed	0.443999657	816	110.74	0.37	0.80	0.20	0.80	0.47
breast_3+Ilastik	0.357504319	1580	141.37	0.59	0.99	0.00	0.99	0.91
breast_3+Ilastik_bad	0.416161311	1019	52.48	0.38	0.48	0.52	0.48	0.73
breast_3+StarDist	0.445971695	1389	147.36	0.51	0.99	0.00	0.99	0.99
breast_3+StarDist_bad	0.445955033	1369	121.42	0.51	0.99	0.00	0.99	0.99
breast_3+Cellpose	0.445147031	881	132.22	0.33	0.99	0.00	0.99	0.63
breast_3+Deepcell	0.445174992	955	96.74	0.35	0.82	0.18	0.82	0.69

breast_3+Watershed	0.446076293	1033	102.41	0.38	0.94	0.06	0.94	0.74
breast_4+Ilastik	0.38660926	286	159.36	0.22	0.99	0.00	0.99	0.97
breast_4+Ilastik_bad	0.385033363	296	75.83	0.22	0.79	0.21	0.79	0.99
breast_4+StarDist	0.446044862	102	1268.06	0.08	0.99	0.00	0.99	0.34
breast_4+StarDist_bad	0.445336359	256	193.98	0.19	0.99	0.00	0.99	0.86
breast_4+Cellpose	0.445395022	187	124.85	0.14	0.83	0.17	0.83	0.63
breast_4+Deepcell	0.445310104	203	115.70	0.15	0.83	0.17	0.83	0.69
breast_4+Watershed	0.445204229	218	109.35	0.16	0.84	0.16	0.84	0.17
breast_5+Ilastik	0.359042576	2159	116.29	0.76	0.99	0.00	0.99	0.80
breast_5+Ilastik_bad	0.370763565	2682	63.27	0.95	0.99	0.00	0.99	0.99
breast_5+StarDist	0.44659975	1376	178.89	0.49	0.99	0.00	0.99	0.51
breast_5+StarDist_bad	0.446418999	1345	132.44	0.47	0.99	0.00	0.99	0.50
breast_5+Cellpose	0.444480998	1277	123.31	0.45	0.99	0.00	0.99	0.48
breast_5+Deepcell	0.444502651	1357	102.21	0.48	0.93	0.07	0.93	0.51
breast_5+Watershed	0.446527401	1227	113.72	0.43	0.94	0.06	0.94	0.46
breast_6+Ilastik	0.381668883	578	149.89	0.40	0.99	0.00	0.99	0.69
breast_6+Ilastik_bad	0.403431887	838	61.56	0.58	0.99	0.00	0.99	0.99
breast_6+StarDist	0.447038243	333	211.88	0.23	0.99	0.00	0.99	0.40
breast_6+StarDist_bad	0.446905353	447	127.03	0.31	0.99	0.00	0.99	0.53
breast_6+Cellpose	0.445334633	244	117.37	0.17	0.61	0.39	0.61	0.29
breast_6+Deepcell	0.445417173	361	98.04	0.25	0.75	0.25	0.75	0.43
breast_6+Watershed	0.446865394	375	101.70	0.26	0.81	0.19	0.81	0.45
breast_7+Ilastik	0.387198498	1398	129.79	0.60	0.99	0.00	0.99	0.99
breast_7+Ilastik_bad	0.390747581	1339	97.29	0.57	0.99	0.00	0.99	0.96
breast_7+StarDist	0.447125445	1304	136.16	0.56	0.99	0.00	0.99	0.93
breast_7+StarDist_bad	0.447126421	1202	128.02	0.51	0.99	0.00	0.99	0.86
breast_7+Cellpose	0.445547173	1099	104.69	0.47	0.99	0.00	0.99	0.79
breast_7+Deepcell	0.445307077	960	94.10	0.41	0.96	0.04	0.96	0.69
breast_7+Watershed	0.447094318	960	107.44	0.41	0.99	0.00	0.99	0.69

breast_8+Ilastik	0.424121776	954	149.45	0.41	0.99	0.00	0.99	0.65
breast_8+Ilastik_bad	0.434159098	1471	58.80	0.63	0.99	0.00	0.99	0.99
breast_8+StarDist	0.446061915	563	208.20	0.24	0.99	0.00	0.99	0.38
breast_8+StarDist_bad	0.446045291	716	143.22	0.31	0.99	0.00	0.99	0.49
breast_8+Cellpose	0.445621683	485	128.33	0.21	0.99	0.00	0.99	0.33
breast_8+Deepcell	0.445438594	540	138.33	0.23	0.99	0.00	0.99	0.37
breast_8+Watershed	0.446223041	552	107.74	0.24	0.99	0.00	0.99	0.38
breast_9+Ilastik	0.361659816	1551	136.92	0.63	0.99	0.00	0.99	0.99
breast_9+Ilastik_bad	0.4110473	1279	129.38	0.52	0.99	0.00	0.99	0.82
breast_9+StarDist	0.446118262	1104	190.35	0.45	0.99	0.00	0.99	0.71
breast_9+StarDist_bad	0.446309871	1197	127.86	0.48	0.99	0.00	0.99	0.77
breast_9+Cellpose	0.445761043	1014	129.15	0.41	0.99	0.00	0.99	0.65
breast_9+Deepcell	0.445844391	1127	126.40	0.46	0.99	0.00	0.99	0.73
breast_9+Watershed	0.446361463	1007	108.55	0.41	0.96	0.04	0.96	0.65
breast_10+Ilastik	0.386080898	1119	73.42	0.43	0.99	0.00	0.99	0.99
breast_10+Ilastik_bad	0.425545142	232	108.66	0.09	0.36	0.64	0.36	0.21
breast_10+StarDist	0.44522754	137	119.98	0.05	0.23	0.77	0.23	0.12
breast_10+StarDist_bad	0.442215026	91	1723.89	0.03	0.99	0.00	0.99	0.08
breast_10+Cellpose	0.445554302	138	117.28	0.05	0.23	0.77	0.23	0.12
breast_10+Deepcell	0.4456588	141	113.87	0.05	0.23	0.77	0.23	0.13
breast_10+Watershed	0.445162532	159	105.47	0.06	0.24	0.76	0.24	0.14
chl_1+Ilastik	0.352647089	3493	70.37	1.40	0.99	0.00	0.99	0.60
chl_1+Ilastik_bad	0.353638585	2027	108.66	0.81	0.99	0.00	0.99	0.35
chl_1+StarDist	0.353660913	1470	164.27	0.59	0.99	0.00	0.99	0.25
chl_1+StarDist_bad	0.35429835	846	103.41	0.34	0.44	0.56	0.44	0.15
chl_1+Cellpose	0.354307748	1099	88.61	0.44	0.49	0.51	0.49	0.19
chl_1+Deepcell	0.354852401	41	136.73	0.02	0.03	0.97	0.03	0.01
chl_1+Watershed	0.350463483	5828	58.53	2.33	0.99	0.00	0.99	0.99
chl_2+Ilastik	0.35397614	2867	82.34	1.15	0.99	0.00	0.99	0.57

chl_2+Ilastik_bad	0.354197814	1663	115.62	0.67	0.99	0.00	0.99	0.33
chl_2+StarDist	0.354461315	1504	157.41	0.60	0.99	0.00	0.99	0.30
chl_2+StarDist_bad	0.354517022	1377	96.42	0.55	0.71	0.29	0.71	0.27
chl_2+Cellpose	0.35431009	1470	94.11	0.59	0.74	0.26	0.74	0.29
chl_2+Deepcell	0.355444047	111	87.14	0.04	0.05	0.95	0.05	0.02
chl_2+Watershed	0.353783604	5044	63.36	2.02	0.99	0.00	0.99	0.99
chl_3+Ilastik	0.351348106	3618	68.16	1.45	0.99	0.00	0.99	0.99
chl_3+Ilastik_bad	0.352087391	2372	89.60	0.95	0.99	0.00	0.99	0.66
chl_3+StarDist	0.3528963	1573	154.41	0.63	0.99	0.00	0.99	0.43
chl_3+StarDist_bad	0.353558132	593	121.67	0.24	0.37	0.63	0.37	0.16
chl_3+Cellpose	0.353391329	782	74.53	0.31	0.30	0.70	0.30	0.22
chl_3+Deepcell	0.353270882	31	97.10	0.01	0.02	0.98	0.02	0.01
chl_3+Watershed	0.352311934	3049	56.54	1.22	0.88	0.12	0.88	0.84
rln_1+Ilastik	0.381390872	2761	89.09	1.10	0.99	0.00	0.99	0.99
rln_1+Ilastik_bad	0.382811103	1841	130.53	0.74	0.99	0.00	0.99	0.67
rln_1+StarDist	0.383960875	1310	184.43	0.52	0.99	0.00	0.99	0.47
rln_1+StarDist_bad	0.385403435	835	115.12	0.33	0.46	0.54	0.46	0.30
rln_1+Cellpose	0.386955131	308	85.83	0.12	0.13	0.87	0.13	0.11
rln_1+Deepcell	0.383744551	1651	140.55	0.66	0.99	0.00	0.99	0.60
rln_1+Watershed	0.383957964	2728	64.64	1.09	0.85	0.15	0.85	0.99
rln_2+Ilastik	0.382028503	3668	67.13	1.47	0.99	0.00	0.99	0.99
rln_2+Ilastik_bad	0.383229435	2033	120.33	0.81	0.99	0.00	0.99	0.55
rln_2+StarDist	0.383685476	1335	169.02	0.53	0.99	0.00	0.99	0.36
rln_2+StarDist_bad	0.384854987	195	224.65	0.08	0.22	0.78	0.22	0.05
rln_2+Cellpose	0.382704408	6	59.58	0.00	0.00	1.00	0.00	0.00
rln_2+Deepcell	0.383966561	1243	116.17	0.50	0.72	0.28	0.72	0.34
rln_2+Watershed	0.382673558	3481	49.46	1.39	0.86	0.14	0.86	0.95
rln_3+Ilastik	0.372575251	2914	83.92	1.17	0.99	0.00	0.99	0.99
rln_3+Ilastik_bad	0.377590649	1957	123.03	0.78	0.99	0.00	0.99	1.00

rln_3+StarDist	0.381952096	1303	179.12	0.52	0.99	0.00	0.99	0.45
rln_3+StarDist_bad	0.385896197	656	138.88	0.26	0.49	0.51	0.49	0.23
rln_3+Cellpose	0.386744646	147	75.73	0.06	0.06	0.94	0.06	0.05
rln_3+Deepcell	0.381020508	1528	133.91	0.61	0.99	0.00	0.99	0.52
rln_3+Watershed	0.374561184	2863	61.46	1.15	0.94	0.06	0.94	0.98
tonsil_1+Ilastik	0.456536356	3219	74.96	1.29	0.99	0.00	0.99	0.99
tonsil_1+Ilastik_bad	0.45741031	1420	165.31	0.57	0.99	0.00	0.99	0.44
tonsil_1+StarDist	0.457283437	1640	141.73	0.66	0.99	0.00	0.99	0.51
tonsil_1+StarDist_bad	0.457728962	881	91.42	0.35	0.50	0.50	0.50	0.27
tonsil_1+Cellpose	0.458559147	341	85.48	0.14	0.18	0.82	0.18	0.11
tonsil_1+Deepcell	0.457153493	1366	118.08	0.55	1.00	0.00	1.00	0.42
tonsil_1+Watershed	0.456635468	2788	57.75	1.12	0.99	0.01	0.99	0.87
tonsil_2+Ilastik	0.457046172	3201	77.57	1.28	0.99	0.00	0.99	0.99
tonsil_2+Ilastik_bad	0.457296808	1586	153.50	0.63	0.99	0.00	0.99	0.50
tonsil_2+StarDist	0.457004442	1717	138.66	0.69	0.99	0.00	0.99	0.54
tonsil_2+StarDist_bad	0.457555478	861	92.10	0.34	0.51	0.49	0.51	0.27
tonsil_2+Cellpose	0.457515508	1398	77.95	0.56	0.70	0.30	0.70	0.44
tonsil_2+Deepcell	0.457157033	1625	116.27	0.65	0.99	0.00	0.99	0.51
tonsil_2+Watershed	0.457011919	3027	55.83	1.21	0.99	0.00	0.99	0.95
tonsil_3+Ilastik	0.456411392	3245	76.80	1.30	0.99	0.00	0.99	0.99
tonsil_3+Ilastik_bad	0.456372844	1662	135.31	0.66	0.99	0.00	0.99	0.51
tonsil_3+StarDist	0.456626985	1643	143.51	0.66	0.99	0.00	0.99	0.51
tonsil_3+StarDist_bad	0.456705077	644	102.46	0.26	0.38	0.62	0.38	0.20
tonsil_3+Cellpose	0.457036518	190	77.99	0.08	0.08	0.92	0.08	0.06
tonsil_3+Deepcell	0.457041617	1533	133.08	0.61	0.99	0.00	0.99	0.47
tonsil_3+Watershed	0.456444611	3204	52.34	1.28	0.96	0.04	0.96	0.99

Batch	Fraction of Extracellular Foreground Pixel Variation	Marker Intensity Variation	Reciprocal of 1+ Marker Intensity Variation	Fraction of First PC of Marker Intensity Variation	Silhouette Score Homogeneity	Cell Size Standard Deviation	Reciprocal of 1+ Log Cell Size Standard Deviation	Cell Variance by Cluster
breast_1+Ilastik	0.25	0.77	0.56	0.55	0.16	59.09	0.2	0.5
breast_1+Ilastik_bad	0.11	0.94	0.52	0.5	0.22	41.93	0.21	0.64
breast_1+StarDist	0.26	-1.86	-1.17	0.72	0.26	113.74	0.17	0.56
breast_1+StarDist_bad	0.1	-1.98	-1.02	0.69	0.32	167.15	0.16	0.63
breast_1+Cellpose	0.06	-0.87	7.72	0.47	0.27	43.25	0.21	0.33
breast_1+Deepcell	0.1	-0.85	6.83	0.44	0.22	49.3	0.2	0.53
breast_1+Watershed	0.16	-1.57	-1.74	0.67	0.32	70.41	0.19	0.57
breast_2+Ilastik	0.44	0.65	0.61	0.74	0.18	59.7	0.2	0.46
breast_2+Ilastik_bad	0.25	0.83	0.55	0.62	0.24	26.44	0.23	0.69
breast_2+StarDist	0.2	4.25	0.19	0.67	0.29	53.98	0.2	0.43
breast_2+StarDist_bad	0.14	15.36	0.06	0.7	0.19	58.07	0.2	0.42
breast_2+Cellpose	0.08	-1.8	-1.25	0.46	0.21	31.74	0.22	0.45
breast_2+Deepcell	0.21	-3.35	-0.42	0.46	0.08	34.93	0.22	0.43
breast_2+Watershed	0.13	0.81	0.55	0.68	0.18	68.4	0.19	0.6
breast_3+Ilastik	0.73	1.07	0.48	0.74	0.22	64.58	0.19	0.45
breast_3+Ilastik_bad	0.36	1.16	0.46	0.69	0.3	28.76	0.23	0.58
breast_3+StarDist	0.48	0.13	0.88	0.58	0.15	103.83	0.18	0.73
breast_3+StarDist_bad	0.27	0.01	0.99	0.57	0.18	60.84	0.2	0.47
breast_3+Cellpose	0.02	2.62	0.28	0.65	0.16	68.12	0.19	0.5
breast_3+Deepcell	0.11	-2.08	-0.92	0.62	0.14	55.55	0.2	0.56
breast_3+Watershed	0.03	-0.04	1.04	0.58	0.25	52.04	0.2	0.49
breast_4+Ilastik	0.54	0.77	0.56	0.88	0.43	86.64	0.18	0.52
breast_4+Ilastik_bad	0.18	0.77	0.56	0.85	0.33	50.78	0.2	0.66
breast_4+StarDist	2.43	-1.78	-1.27	0.68	0.38	1453.47	0.12	0.98

breast_4+StarDist_bad	0.53	-16.7	-0.06	0.7	0.44	126.21	0.17	0.55
breast_4+Cellpose	0.11	-0.67	3.04	0.62	0.14	65.71	0.19	0.47
breast_4+Deepcell	0.1	-0.6	2.49	0.6	0.2	71.88	0.19	0.58
breast_4+Watershed	0.11	-2.27	-0.79	0.7	0.41	52.15	0.2	0.44
breast_5+Ilastik	0.53	0.88	0.53	0.76	0.48	49.63	0.2	0.48
breast_5+Ilastik_bad	0.11	0.87	0.54	0.76	0.28	38.69	0.21	0.66
breast_5+StarDist	0.55	4.29	0.19	0.84	0.48	87.72	0.18	0.49
breast_5+StarDist_bad	0.17	14.71	0.06	0.83	0.52	73.5	0.19	0.48
breast_5+Cellpose	0.03	1.69	0.37	0.56	0.17	53.25	0.2	0.48
breast_5+Deepcell	0.04	11.94	0.08	0.57	0.21	44.01	0.21	0.42
breast_5+Watershed	0.05	-8.03	-0.14	0.81	0.48	64.66	0.19	0.56
breast_6+Ilastik	0.31	0.83	0.55	0.37	0.32	71.86	0.19	0.48
breast_6+Ilastik_bad	0.04	1	0.5	0.38	0.29	44.12	0.21	0.68
breast_6+StarDist	0.26	-6.04	-0.2	0.52	0.3	228.75	0.16	0.93
breast_6+StarDist_bad	0.1	-3.86	-0.35	0.5	0.31	67.09	0.19	0.52
breast_6+Cellpose	0.22	-0.54	2.17	0.57	0.22	52.65	0.2	0.43
breast_6+Deepcell	0.14	9.38	0.1	0.55	0.22	54.97	0.2	0.52
breast_6+Watershed	0.1	-7.51	-0.15	0.52	0.3	55.1	0.2	0.51
breast_7+Ilastik	0.5	1.09	0.48	0.54	0.42	55.96	0.2	0.46
breast_7+Ilastik_bad	0.2	1.17	0.46	0.52	0.43	45.14	0.21	0.49
breast_7+StarDist	0.5	-2.57	-0.64	0.57	0.36	57.66	0.2	0.46
breast_7+StarDist_bad	0.36	-2.48	-0.68	0.56	0.44	51.56	0.2	0.43
breast_7+Cellpose	0.1	-0.91	11.59	0.47	0.19	43.93	0.21	0.44
breast_7+Deepcell	0.02	-0.81	5.24	0.49	0.25	43.21	0.21	0.51
breast_7+Watershed	0.06	-3.66	-0.38	0.61	0.43	59.37	0.2	0.61
breast_8+Ilastik	0.65	0.81	0.55	0.43	0.2	74.44	0.19	0.49
breast_8+Ilastik_bad	0.21	0.95	0.51	0.4	0.18	71.51	0.19	0.95
breast_8+StarDist	0.47	9.93	0.09	0.44	0.16	238.08	0.15	0.86
breast_8+StarDist_bad	0.33	-23.74	-0.04	0.42	0.16	100.97	0.18	0.59

breast_8+Cellpose	0.04	2.43	0.29	0.38	0.2	61.17	0.2	0.43
breast_8+Deepcell	0.12	3.27	0.23	0.38	0.19	88.64	0.18	0.5
breast_8+Watershed	0.02	0.99	0.5	0.38	0.19	62.63	0.19	0.57
breast_9+Ilastik	0.53	0.65	0.61	0.61	0.15	63.86	0.19	0.5
breast_9+Ilastik_bad	0.3	0.62	0.62	0.65	0.28	67.21	0.19	0.5
breast_9+StarDist	0.38	2.71	0.27	0.45	0.17	114.38	0.17	0.55
breast_9+StarDist_bad	0.15	4.1	0.2	0.44	0.08	56.99	0.2	0.45
breast_9+Cellpose	0.09	0.65	0.61	0.58	0.21	52.09	0.2	0.44
breast_9+Deepcell	0.15	0.65	0.61	0.58	0.22	57.31	0.2	0.46
breast_9+Watershed	0.02	3.05	0.25	0.43	0.2	55.21	0.2	0.51
breast_10+Ilastik	0.14	0.98	0.5	0.86	0.19	55.6	0.2	0.77
breast_10+Ilastik_bad	0.53	0.73	0.58	0.82	0.19	138.12	0.17	0.86
breast_10+StarDist	0.41	-0.63	2.73	0.53	0.14	33.87	0.22	0.29
breast_10+StarDist_bad	0.97	-0.01	1.01	0.8	0.24	1565.04	0.12	0.8
breast_10+Cellpose	0.33	0.72	0.58	0.43	0.35	46.46	0.21	0.22
breast_10+Deepcell	0.29	7.66	0.12	0.38	0.22	57.18	0.2	0.45
breast_10+Watershed	0.44	0.61	0.62	0.57	0.36	36.15	0.22	0.26
chl_1+Ilastik	0.11	-3.41	-0.41	0.43	0.17	30.22	0.23	0.43
chl_1+Ilastik_bad	0.05	-3.74	-0.36	0.43	0.17	51.4	0.2	0.49
chl_1+StarDist	0.11	-1.32	-3.15	0.48	0.06	97.06	0.18	0.61
chl_1+StarDist_bad	0.22	0.12	0.89	0.4	0.13	57.15	0.2	0.48
chl_1+Cellpose	0.2	-3.86	-0.35	0.4	0.15	33.55	0.22	0.4
chl_1+Deepcell	0.48	0.66	0.6	0.5	0.22	172.05	0.16	0.61
chl_1+Watershed	0.29	0.1	0.91	0.39	0.16	34.13	0.22	0.58
chl_2+Ilastik	0.1	12.17	0.08	0.4	0.11	35.4	0.22	0.43
chl_2+Ilastik_bad	0.01	1.04	0.49	0.4	0.11	54.99	0.2	0.48
chl_2+StarDist	0.11	2.65	0.27	0.4	0.11	79.89	0.19	0.5
chl_2+StarDist_bad	0.11	17.37	0.05	0.38	0.14	34.86	0.22	0.38
chl_2+Cellpose	0.1	-5.48	-0.22	0.38	0.12	36.89	0.22	0.39

chl_2+Deepcell	0.36	-3.96	-0.34	0.37	0.04	118.1	0.17	0.78
chl_2+Watershed	0.26	-6.76	-0.17	0.37	0.08	34.05	0.22	0.54
chl_3+Ilastik	0.11	-3.19	-0.46	0.41	0.11	27.4	0.23	0.42
chl_3+Ilastik_bad	0.03	-2.91	-0.52	0.43	0.07	40.53	0.21	0.46
chl_3+StarDist	0.11	-3.74	-0.37	0.47	0.09	80.91	0.19	0.51
chl_3+StarDist_bad	0.28	12.98	0.07	0.44	0.12	118.96	0.17	0.69
chl_3+Cellpose	0.29	1.57	0.39	0.42	0.09	29	0.23	0.4
chl_3+Deepcell	0.52	2.38	0.3	0.53	0.09	101.45	0.18	0.46
chl_3+Watershed	0.05	-2.65	-0.61	0.42	0.09	32.49	0.22	0.59
rln_1+Ilastik	0.09	-39.7	-0.03	0.47	0.12	38.53	0.21	0.44
rln_1+Ilastik_bad	0.07	86.65	0.01	0.47	0.19	72.33	0.19	0.58
rln_1+StarDist	0.08	-6.28	-0.19	0.49	0.17	97.98	0.18	0.54
rln_1+StarDist_bad	0.27	-0.42	1.73	0.51	0.13	46.54	0.21	0.42
rln_1+Cellpose	0.45	2.94	0.25	0.52	0.16	31.88	0.22	0.35
rln_1+Deepcell	0.06	-109.64	-0.01	0.48	0.21	53.24	0.2	0.38
rln_1+Watershed	0.07	-4.13	-0.32	0.43	0.14	37.39	0.22	0.58
rln_2+Ilastik	0.09	3.92	0.2	0.38	0.12	30.24	0.23	0.45
rln_2+Ilastik_bad	0.09	3.47	0.22	0.4	0.2	70.18	0.19	0.58
rln_2+StarDist	0.05	2.45	0.29	0.43	0.15	95.37	0.18	0.56
rln_2+StarDist_bad	0.36	-1.71	-1.41	0.46	0.17	211.41	0.16	0.63
rln_2+Cellpose	0.62	-383.78	0	0.62	0.16	21.39	0.25	0.04
rln_2+Deepcell	0.11	-1.3	-3.38	0.4	0.17	59.18	0.2	0.49
rln_2+Watershed	0.05	4.19	0.19	0.36	0.14	32.75	0.22	0.63
rln_3+Ilastik	0.17	-0.76	4.23	0.56	0.13	37.85	0.22	0.44
rln_3+Ilastik_bad	0.17	-0.72	3.58	0.61	0.17	68.49	0.19	0.57
rln_3+StarDist	0.16	-0.69	3.24	0.64	0.12	97.31	0.18	0.51
rln_3+StarDist_bad	0.26	-0.79	4.85	0.5	0.2	111.16	0.18	0.73
rln_3+Cellpose	0.46	-0.86	7.34	0.49	0.23	29.89	0.23	0.36
rln_3+Deepcell	0.05	-0.68	3.12	0.61	0.12	53.5	0.2	0.41

rln_3+Watershed	0.03	-0.78	4.46	0.53	0.09	40.57	0.21	0.65
tonsil_1+Ilastik	0.19	0.64	0.61	0.39	0.22	35.39	0.22	0.47
tonsil_1+Ilastik_bad	0.21	1.56	0.39	0.47	0.22	102.73	0.18	0.55
tonsil_1+StarDist	0.17	0.62	0.62	0.4	0.23	73.69	0.19	0.51
tonsil_1+StarDist_bad	0.23	3.7	0.21	0.46	0.23	34.83	0.22	0.37
tonsil_1+Cellpose	0.38	-9.6	-0.12	0.47	0.21	39.75	0.21	0.45
tonsil_1+Deepcell	0	0.19	0.84	0.38	0.18	56.23	0.2	0.48
tonsil_1+Watershed	0	0.53	0.66	0.37	0.23	34.96	0.22	0.58
tonsil_2+Ilastik	0.27	4.6	0.18	0.45	0.25	30.5	0.23	0.39
tonsil_2+Ilastik_bad	0.26	2.09	0.32	0.45	0.2	97.68	0.18	0.64
tonsil_2+StarDist	0.24	3.74	0.21	0.44	0.23	64.39	0.19	0.46
tonsil_2+StarDist_bad	0.2	2.08	0.33	0.41	0.18	38.02	0.22	0.43
tonsil_2+Cellpose	0.12	24.42	0.04	0.4	0.29	30.87	0.23	0.4
tonsil_2+Deepcell	0.1	4.91	0.17	0.44	0.17	49.06	0.2	0.41
tonsil_2+Watershed	0.04	5.02	0.17	0.45	0.22	30.8	0.23	0.55
tonsil_3+Ilastik	0.19	11.66	0.08	0.44	0.28	32.17	0.22	0.42
tonsil_3+Ilastik_bad	0.14	4.83	0.17	0.47	0.31	81.94	0.18	0.55
tonsil_3+StarDist	0.17	8.14	0.11	0.48	0.31	65.68	0.19	0.46
tonsil_3+StarDist_bad	0.32	4.72	0.17	0.52	0.21	56.59	0.2	0.52
tonsil_3+Cellpose	0.4	-1.67E+15	0	0.43	0.26	28.77	0.23	0.36
tonsil_3+Deepcell	0.08	10.19	0.09	0.46	0.32	59.95	0.2	0.45
tonsil_3+Watershed	0.02	15.13	0.06	0.44	0.26	28.73	0.23	0.53

3.2 Supplementary Table 2. Pairwise comparison of segmentation performance of three representative methods as well as controls on two methods. All p-values are computed using the Student's t test. A p-value <0.05 is considered significant.

Exp	Ilastik: Watershed	Ilastik: Stardist	Ilastik: Cellpose	Ilastik: Deepcell	Watershed: Stardist	Watershed: Cellpose	Watershed: Deepcell	Stardist: Cellpose	Stardist: Deepcell
breast_1	0.00E+00	0.00E+00	0.00E+00	0.00E+00	7.20E-01	1.10E-07	1.20E-02	8.30E-09	3.60E-03
breast_2	3.30E-08	6.40E-12	4.50E-06	7.30E-05	3.20E-01	5.90E-23	5.70E-19	1.70E-31	3.90E-25
breast_3	0.00E+00	0.00E+00	0.00E+00	0.00E+00	1.70E-01	3.50E-22	4.10E-21	3.70E-20	3.10E-19
breast_4	6.00E-161	6.00E-96	1.60E-144	4.80E-153	6.60E-04	3.80E-01	6.10E-01	2.20E-02	7.10E-03
breast_5	0.00E+00	0.00E+00	0.00E+00	0.00E+00	4.90E-01	3.00E-53	5.80E-52	1.00E-66	6.20E-65
breast_6	1.40E-137	1.40E-125	2.00E-93	1.70E-129	5.10E-01	9.80E-08	2.60E-09	3.50E-17	6.60E-20
breast_7	0.00E+00	0.00E+00	0.00E+00	0.00E+00	7.70E-01	4.50E-36	7.00E-40	4.00E-47	5.40E-52
breast_8	1.10E-84	4.50E-85	6.40E-72	5.60E-78	2.50E-01	3.10E-06	1.30E-08	8.90E-04	9.20E-06
breast_9	0.00E+00	0.00E+00	0.00E+00	0.00E+00	4.50E-02	2.20E-08	4.70E-07	1.30E-03	1.00E-02
breast_10	1.10E-81	6.40E-72	4.40E-73	1.10E-74	8.30E-01	1.50E-01	5.10E-02	2.70E-01	1.20E-01
chl_1	1.40E-55	4.40E-12	1.40E-24	4.30E-03	6.50E-59	3.30E-65	1.00E-04	4.10E-05	6.70E-02
chl_2	5.00E-02	8.30E-05	6.80E-03	1.20E-04	3.80E-08	2.10E-05	6.00E-05	2.60E-01	5.70E-03
chl_3	5.30E-13	1.60E-21	1.40E-21	6.10E-02	1.20E-04	2.90E-08	3.00E-01	8.10E-03	6.50E-01
rln_1	1.10E-23	6.70E-15	7.90E-19	1.50E-14	9.90E-01	1.80E-11	3.70E-01	4.50E-13	4.30E-01
rln_2	9.20E-07	1.40E-20	7.80E-01	1.60E-26	2.90E-10	9.90E-01	2.10E-15	5.90E-01	9.90E-02
rln_3	1.50E-05	2.20E-71	5.00E-22	4.60E-64	1.10E-47	1.40E-17	3.00E-40	7.70E-11	1.10E-02
tonsil_1	3.60E-01	1.30E-09	4.20E-18	3.10E-06	2.70E-07	2.30E-16	1.30E-04	1.00E-08	3.60E-01
tonsil_2	7.70E-01	7.60E-01	1.20E-03	4.30E-01	9.60E-01	6.60E-04	3.10E-01	2.20E-03	3.50E-01
tonsil_3	8.00E-01	1.70E-01	1.00E-01	7.30E-05	2.50E-01	1.20E-01	1.80E-04	3.00E-01	2.30E-02

Exp	Cellpose: Deepcell	Ilastik: Ilastik-Poor	Stardist: Stardist-Poor
breast_1	1.50E-02	1.20E-06	6.90E-02
breast_2	7.20E-01	2.90E-236	5.50E-02
breast_3	8.00E-01	1.90E-281	8.20E-01
breast_4	7.20E-01	3.70E-01	4.80E-03
breast_5	8.80E-01	1.20E-53	1.20E-01
breast_6	6.00E-01	4.60E-27	5.70E-01
breast_7	6.40E-02	2.60E-03	9.90E-01
breast_8	1.50E-01	3.60E-35	9.00E-01
breast_9	3.60E-01	7.70E-288	1.00E-01
breast_10	6.60E-01	1.60E-53	2.60E-07
chl_1	3.60E-01	3.20E-14	1.90E-04
chl_2	1.20E-03	6.20E-02	6.70E-01
chl_3	8.60E-01	5.30E-07	1.60E-03
rln_1	9.60E-13	2.30E-06	4.50E-07
rln_2	4.60E-01	5.70E-15	4.80E-04
rln_3	8.40E-11	2.30E-26	3.60E-26
tonsil_1	1.10E-09	1.20E-11	5.00E-03
tonsil_2	3.10E-02	7.20E-02	3.90E-03
tonsil_3	9.90E-01	8.00E-01	7.50E-01

3.3 Supplementary Table 3. Comparison of various existing segmentation metrics with entropy from ESQmodel. Spearman's rank correlation coefficient was calculated for each pair of metrics across tissue types.

Batch	Cell Coverage by Hundred Pixels Correlation	Cell Foreground Coverage Correlation	Cell Background Coverage Correlation	Cell Mask Foreground Coverage Correlation	Cell-Nuclei Match Correlation	Fraction of Extracellular Foreground Pixel Variation Correlation	Marker Intensity Variation Correlation
breast	0.267	0.894	0.894	0.894	0.013	0.160	0.006
chl	0.008	0.070	0.070	0.070	0.002	0.427	0.775
rln	0.001	0.002	0.002	0.002	0.000	0.047	0.594
tonsil	0.001	0.139	0.139	0.139	0.003	0.186	0.131

Batch	Reciprocal of 1+ Marker Intensity Variation Correlation	Fraction of First PC of Marker Intensity Variatio Correlationn	Silhouette Score Homogeneity Correlation	Cell Size Standard Deviation Correlation	Reciprocal of 1+ Log Cell Size Standard Deviation Correlation	Cell Variance by Cluster Correlation
breast	0.003	0.241	0.497	0.097	0.097	0.804
chl	0.163	0.103	0.529	0.053	0.053	0.991
rln	0.375	0.241	0.005	0.646	0.646	0.679
tonsil	0.683	0.947	0.029	0.654	0.654	0.139

3.4 Supplementary Table 4. Scores of entropy from ESQmodel and various existing segmentation metrics to complement segmentation quality assessment for tonsil datasets across different imaging platforms.

Batch	Entropy	Cell Count	Average Cell Size	Cell Coverage by Hundred Pixels	Cell Foreground Coverage	Cell Background Coverage	Cell Mask Foreground Coverage	Cell-Nuclei Match
imc_1+Watershed	0.544860181	4780	112.49	0.48	0.99	0.00	0.99	0.96
imc_1+StarDist	0.54287178	4967	165.40	0.50	0.99	0.00	0.99	0.99
imc_1+Cellpose	0.545094431	466	90.09	0.05	0.08	0.92	0.08	0.09
imc_1+Deepcell	0.54438818	3164	96.16	0.32	0.57	0.43	0.57	0.64
mibi_1+Watershed	0.489011654	7780	52.90	0.19	0.21	0.79	0.21	0.71
mibi_1+StarDist	0.37423464	10903	55.83	0.26	0.31	0.69	0.31	0.99
mibi_1+Cellpose	0.549084992	3089	75.35	0.07	0.12	0.88	0.12	0.28
mibi_1+Deepcell	0.468595464	2591	45.76	0.14	0.14	0.86	0.14	0.24
codex_1+Watershed	0.495657258	29762	49.70	0.25	0.21	0.79	0.21	0.99
codex_1+StarDist	0.49324507	29395	65.67	0.24	0.27	0.73	0.27	0.99
codex_1+Cellpose	0.532136983	9841	61.80	0.08	0.08	0.92	0.08	0.33
codex_1+Deepcell	0.468408897	6026	45.76	0.05	0.04	0.96	0.04	0.20
cycif_1+Watershed	0.434670106	11938	23.86	2.25	0.99	0.00	0.99	0.99
cycif_1+StarDist	0.54146003	533	642.65	0.10	0.99	0.00	0.99	0.04
cycif_1+Cellpose	0.544398863	14	312.56	0.00	0.02	0.98	0.02	0.00
cycif_1+Deepcell	0.500677587	5667	257.82	1.07	0.99	0.00	0.99	0.47

Batch	Fraction of Extracellular Foreground Pixel Variation	Marker Intensity Variation	Reciprocal of 1+ Marker Intensity Variation	Fraction of First PC of Marker Intensity Variation	Silhouette Score Homogeneity	Cell Size Standard Deviation	Reciprocal of 1+ Log Cell Size Standard Deviation	Cell Variance by Cluster
imc_1+Watershed	0.01	1.07	0.48	0.96	0.46	91.73	0.18	0.79
imc_1+StarDist	0.53	0.93	0.52	0.97	0.38	90.08	0.18	0.52
imc_1+Cellpose	0.9	0.96	0.51	0.98	-0.02	35.94	0.22	0.41
imc_1+Deepcell	0.42	1.1	0.48	0.97	0.4	57.73	0.2	0.58
mibi_1+Watershed	0.75	1.65	0.38	0.95	0.17	22.66	0.24	0.42
mibi_1+StarDist	0.65	1.67	0.38	0.94	0.17	31.23	0.23	0.56
mibi_1+Cellpose	0.87	12.47	0.07	0.98	0.96	34.58	0.22	0.48
mibi_1+Deepcell	0.78	1.41	0.41	0.9	0.12	22.13	0.24	0.47
codex_1+Watershed	0.65	0.54	0.65	0.82	0.46	23.5	0.24	0.47
codex_1+StarDist	0.6	0.54	0.65	0.82	0.46	69.61	0.19	1.03
codex_1+Cellpose	0.77	0.68	0.59	0.84	0.45	24.26	0.24	0.39
codex_1+Deepcell	0.69	0.8	0.56	0.72	0.21	22.13	0.24	0.49
cycif_1+Watershed	0.24	0.07	0.93	0.9	0.32	17.04	0.26	0.71
cycif_1+StarDist	0.49	0.06	0.94	0.93	0.39	1520.8	0.12	1.67
cycif_1+Cellpose	0.52	0.07	0.93	0.53	0.42	196.69	0.16	0.39
cycif_1+Deepcell	5.4	0.03	0.97	0.99	0.74	110.14	0.18	0.55

4 Author's Contributions

A.R. and E.L. conceived the study; IMC datasets were generated by A.L., D.W.S and C.S.; E.L. implemented the method and performed data analysis; D.L. and W.F. helped segmenting images; computation resources of the IMAXT consortium were employed; E.L. wrote the manuscript with contributions from A.R. and S.A.; and all authors read and approved the final manuscript.

References

[1] Levine, J.H., Simonds, E.F., Bendall, S.C., Davis, K.L., Amir, E.-a.D., Tadmor, M.D., Litvin, O., Fienberg, H.G., Jager, A., Zunder, E.R., Finck, R., Gedman, A.L., Radtke, I., Downing, J.R., Pe'er, D., Nolan, G.P.: Data-driven phenotypic dissection of AML reveals progenitor-like cells that correlate with prognosis. Cell **162**(1), 184–197 (2015)

[2] Ali, H.R., Jackson, H.W., Zanotelli, V.R.T., Danenberg, E., Fischer, J.R., Bardwell, H., Provenzano, E., Team, C.I.G.C., Rueda, O.M., Chin, S.-F., Aparicio, S., Caldas, C., Bodenmiller, B.: Imaging mass cytometry and multiplatform genomics define the phenogenomic landscape of breast cancer. Nature Cancer **1**(1), 163–175 (2020)

[3] Vito RT Zanotelli, B.B.: ImcSegmentationPipeline: A pixel-classification based multiplexed image segmentation pipeline. Zenodo (2022). https://doi.org/10.5281/zenodo.3841961

[4] Rashid, R., Gaglia, G., Chen, Y.-A., Lin, J.-R., Du, Z., Maliga, Z., Schapiro, D., Yapp, C., Muhlich, J., Sokolov, A., Sorger, P., Santagata, S.: Highly multiplexed immunofluorescence imaging of human tissues and tumors using t-cycif and conventional optical microscopes. Scientific Data **6**, 323 (2019)

[5] Berg, S., Kutra, D., Kroeger, T., Straehle, C.N., Kausler, B.X., Haubold, C., Schiegg, M., Ales, J., Beier, T., Rudy, M., Eren, K., Cervantes, J.I., Xu, B., Beuttenmueller, F., Wolny, A., Zhang, C., Koethe, U., Hamprecht, F.A., Kreshuk, A.: ilastik: interactive machine learning for (bio)image analysis. Nature Methods **16**(1), 1226–32 (2019)

[6] Lee, E.: Spatial Proteomics Framework. GitHub (2022). https://github.com/ericlee0920/Spatial-Proteomics-Framework

[7] Stirling, D.R., Swain-Bowden, M.J., Lucas, A.M., Carpenter, A.E., Cimini, B.A., Goodman, A.: Cellprofiler 4: improvements in speed, utility and usability. BMC Bioinformatics **22**(433) (2021)

[8] Bankhead, P., Loughrey, M.B., Fernández, J.A., Dombrowski, Y., McArt, D.G., Dunne, P.D., McQuaid, S., Gray, R.T., Murray, L.J., Coleman, H.G., James, J.A., Salto-Tellez, M., Hamilton, P.W.: Qupath: Open source software for digital pathology image analysis. Scientific Reports **7**(16878) (2017)

[9] STARR: IMC Converter. GitHub (2019). https://github.com/STTARR/imc-converter

[10] Weigert, M., Schmidt, U., Haase, R., Sugawara, K., Myers, G.: Star-convex polyhedra for 3d object detection and segmentation in microscopy. In: The IEEE Winter Conference on Applications of Computer Vision (WACV) (2020).

https://doi.org/10.1109/WACV45572.2020.9093435

[11] Schmidt, U., Weigert, M.: StarDist ImageJ/Fiji Plugin. GitHub (2020). https://github.com/stardist/stardist-imagej/tree/master/src/main/resources/models/2D

[12] Stringer, C., Wang, T., Michaelos, M., Pachitariu, M.: Cellpose: a generalist algorithm for cellular segmentation. Nature Methods **18**(1), 100–6 (2020)

[13] Greenwald, N.F., Miller, G., Moen, E., Kong, A., Kagel, A., Dougherty, T., Fullaway, C.C., McIntosh, B.J., Leow, K.X., Schwartz, M.S., Pavelchek, C., Cui, S., Camplisson, I., Bar-Tal, O., Singh, J., Fong, M., Chaudhry, G., Abraham, Z., Moseley, J., Warshawsky, S., Soon, E., Greenbaum, S., Risom, T., Hollmann, T., Bendall, S.C., Keren, L., Graf, W., Angelo, M., Valen, D.V.: Whole-cell segmentation of tissue images with human-level performance using large-scale data annotation and deep learning. Nature Biotechnology **40**(1), 555–65 (2022)
